# An Efficient and Accurate Iris Recognition Algorithm Based on a Novel Condensed 2-ch Deep Convolutional Neural Network

**DOI:** 10.3390/s21113721

**Published:** 2021-05-27

**Authors:** Guoyang Liu, Weidong Zhou, Lan Tian, Wei Liu, Yingjian Liu, Hanwen Xu

**Affiliations:** School of Microelectronics, Shandong University, Jinan 250100, China; 201712114@mail.sdu.edu.cn (G.L.); tianlan65@sdu.edu.cn (L.T.); davyliu@sdu.edu.cn (W.L.); 201812334@mail.sdu.edu.cn (Y.L.); 201832364@mail.sdu.edu.cn (H.X.)

**Keywords:** iris recognition, online augmentation, convolutional neural network, deep learning, network pruning

## Abstract

Recently, deep learning approaches, especially convolutional neural networks (CNNs), have attracted extensive attention in iris recognition. Though CNN-based approaches realize automatic feature extraction and achieve outstanding performance, they usually require more training samples and higher computational complexity than the classic methods. This work focuses on training a novel condensed 2-channel (2-ch) CNN with few training samples for efficient and accurate iris identification and verification. A multi-branch CNN with three well-designed online augmentation schemes and radial attention layers is first proposed as a high-performance basic iris classifier. Then, both branch pruning and channel pruning are achieved by analyzing the weight distribution of the model. Finally, fast finetuning is optionally applied, which can significantly improve the performance of the pruned CNN while alleviating the computational burden. In addition, we further investigate the encoding ability of 2-ch CNN and propose an efficient iris recognition scheme suitable for large database application scenarios. Moreover, the gradient-based analysis results indicate that the proposed algorithm is robust to various image contaminations. We comprehensively evaluated our algorithm on three publicly available iris databases for which the results proved satisfactory for real-time iris recognition.

## 1. Introduction

Iris texture patterns are believed to be randomly determined during fetal development of the eye and are invariant to age [1]. Hence, the iris pattern of each eye can be seen as a universally unique biometric feature even distinct between twins. As one of the most secure and reliable biometric identification techniques, iris recognition has been widely used in banking, border security control, mobile phones, etc. [2,3,4]. Compared with other mainstream biometric approaches, including face recognition [5], palmprint recognition [6], and fingerprint recognition [7], iris recognition is safer and more sanitary because of its characteristics of being non-contact and having less exposure [8]. The merits of iris recognition have prompted increasing efforts for investigating more accurate and efficient iris feature extraction algorithms under various conditions [9,10,11]. 

Verification and identification are the two main application scenarios for iris recognition. Given an iris image of an eye, the iris recognition system in verification mode will judge whether it is registered or not according to the previously enrolled iris images, which is usually a “one-against-one” comparison scheme. In the identification mode, the system will answer “who is he” to this iris image, which performs a “one-against-all” comparison scheme most of the time.

The deep learning methods, especially convolutional neural network (CNN), have achieved considerable success in many computer vision (CV) tasks [12,13,14,15,16]. Handcrafted feature extraction approaches have been outclassed by CNN, with its capability to automatically learn relevant features from sufficient training data [17,18]. Recent advances in iris recognition have studied the feasibility of applying the CNN to iris image processing, such as iris segmentation [19,20], iris recognition [21,22,23], and fake iris detection [24,25]. Previous studies on iris recognition [21,26] indicated that the CNN-based methods could effectively learn the inherent characteristics of iris images and achieve superior performance than the classic iris matching method represented by IrisCode [27]. The success of early efforts prompts us to further investigate the potential of deep CNN for addressing challenging problems in real-time iris recognition.

## 2. Related Work

The earliest automatic iris recognition system can be traced back to 1987. Flom and Safir [28] proposed a conceptual iris recognition system without implementation details and were authorized the first patent. From then on, various iris texture feature extraction and classification methods emerged, which can be approximately divided into classic handcrafted feature engineering methods and recently appeared deep learning methods.

One of the most influential iris recognition algorithms was proposed by Daugman [27,29,30]. In his pioneering works, the boundary of the pupil and iris was first detected by the integrodifferential operator and normalized by Daugman’s rubber sheet model. Then, the extracted iris was transformed into a series of encoding (which is usually referred to as IrisCode) by applying Gabor phase-quadrant feature descriptor. Finally, in the identification or verification stage, the hamming distance between IrisCode was calculated and hence attained the recognition result. Daugman’s algorithm and workflow are still widely utilized in current iris recognition systems. Later, numerous iris feature extraction approaches arose, including variations of the Gabor kernel [31,32,33], the SIFT and SURF-based features [34,35,36], feature fusion methods [37,38,39], and human-in-the-loop methods [40,41]. These methods usually yield a notable performance with few training data. Nevertheless, their feature extractors are required to be delicately designed and not robust to image contaminations such as eyelid and eyebrow, which places higher demands on image quality and preprocessing steps.

Recently, deep learning-based iris recognition approaches have been increasingly studied. Deep CNN is usually used as a feature extractor, which encodes the iris image to a set of feature vectors and then measures their distance as the aforementioned classic method does. Gangwar et al. [42] proposed a deep CNN model with less risk of overfitting for extracting the iris feature. Nguyen et al. [43] explored the encoding ability of the pre-trained CNN architecture, with results showing that the network, such as AlexNet and VGG-net trained on other large-scale image databases, can be effectively transferred to the task of iris texture feature extraction. Raja et al. [44] extracted robust multi-patch iris features by CNN with sparse filters. More recently, Wang et al. [26] and Liu et al. [45] collected iris features using dilated residual network and capsule network, respectively. In addition, deep CNN can also be directly utilized as a classifier. In this way, the pairwise training dataset was generated with all possible combinations of training samples. In the testing phase, paired images were fed into CNN, and the result was provided to examine whether the images belonged to intra-class or inter-class. With this approach, few training samples were needed for the deep neural network. This type of method was first discussed in detail in the work of Zagoruyko et al. [46], and different types of networks, including siamese, pseudo-siamese, 2-channel (2-ch) deep networks, were constructed for image patch comparison. The experimental results showed that the 2-ch network outperformed the other networks at the cost of computational complexity. Some efforts have also been focused on iris verification using 2-ch network. Liu et al. [47] proposed a 2-ch CNN architecture named DeepIris for heterogeneous iris verification. In their algorithm, six forward propagations were required to prevent rotation differences, which led to the heavy computational burden. Špetlík et al. [48] modified the 2-ch CNN with a unit-circle layer for iris verification. Proença et al. [49] integrated an iris segmentation deep learning model and a 2-ch iris classification CNN for segment-less iris verification.

Though the existing deep learning-based methods are proven to be effective for automatic end-to-end iris feature extraction and classification, several issues remain to be further addressed. For example, due to high computational complexity, the 2-ch methods have only been successfully applied to the iris verification scenario. In addition, the deep learning model is sensitive to image contamination and training data scale, which poses a challenging problem to real-time iris recognition. Furthermore, the hyperparameters in CNN architecture, such as the number of layers and kernels, have not been fully optimized.

The objective of this paper is to develop a deep learning approach with strong robustness to various iris contaminations for large-scale iris identification and verification. To meet this goal and overcome the limitations mentioned above, we construct a multi-branch 2-ch CNN with a radial attention layer. This model is trained with online augmentation schemes to gain a robust iris classifier. Structural pruning is conducted for accurate and efficient iris matching. Finally, the encoding ability of the model is explored, and the performance of iris identification and verification is evaluated on three large-scale databases. The key novelties of our work can be concluded as in the following:

- To allow the 2-ch CNN to apply in the large-scale iris identification scenario, we investigate the encoding ability of the 2-ch CNN in different layers and put forward a hybrid framework which takes advantage of the accurateness of 2-ch CNN and the efficiency of the encoding and matching.
- A radial attention layer that can guide our model to focus on relevant iris regions along the radial direction is proposed, and branch level model pruning is realized by norm computing.
- Three types of online augmentation schemes are designed to enhance the robustness of the model. The successful modeling of brightness jitter, iris image rotation, and radial extension occurring in real-time iris recognition can prevent the model from overfitting and allow the model to train on the small-scale training dataset.
- A condensed 2-ch CNN with optimal architecture is obtained by pruning the model at the channel level as well as the branch level.

The remainder of this paper is organized as follows: Section 3 presents the details of the proposed iris recognition method. Section 4 illustrates the experiment settings and experimental results on three different databases. Section 5 extends the experimental results and makes additional comparisons. Section 6 summarizes the research and draws the conclusion.

## 3. Methods

**Figure 1:**
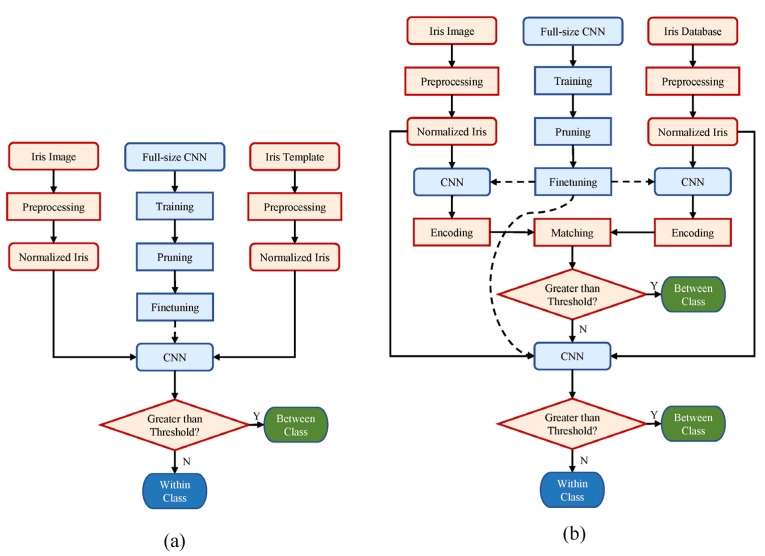
The whole architecture of our iris recognition algorithm. (**a**,**b**) demonstrate the verification and identification workflow, respectively.

The simplified iris identification and verification workflows of the proposed method are illustrated in Figure 1. As shown in Figure 1a, a full-sized CNN is first trained, and the subsequent pruning and finetuning procedures enable the CNN to obtain better performance and efficiency. Finally, a pair of preprocessed and normalized irises is fed into CNN to calculate the inference distance. Meanwhile, the iris identification workflow is illustrated in Figure 1b. Contrary to the “one-against-one” comparison strategy implemented in the verification scenario, the identification scenario needs to conduct more comparison operation in one identification epoch (i.e., the process of a sample paired with all databases). Consequently, we add an external encoding matching step, which can effectively alleviate the computational burden of the 2-ch CNN in iris identification scenario. Further, the details of each step are explained in the following subsections.

### 3.1. Preprocessing

**Figure 2:**
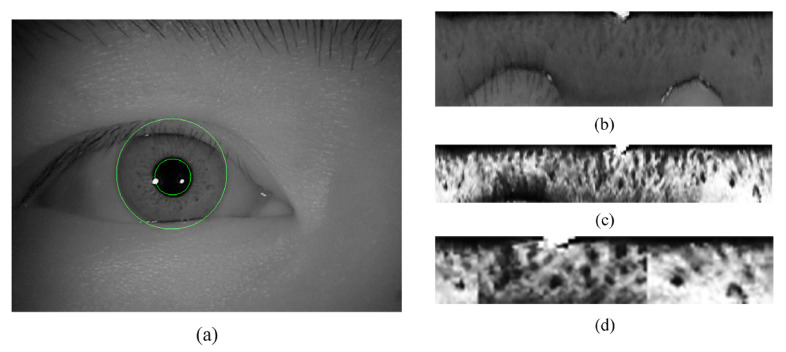
The preprocessing stage including location and segmentation (**a**), normalization (**b**), longitudinal cropping and image enhancement (**c**), and optional horizontal cropping step (**d**). The region between inner and outer green boundary in (**a**) is the segmented iris.

We uniformly perform iris segmentation and normalization using an open-source tool named OSIRIS v4.1 [50]. Figure 2 depicts a sample for each preprocessing step. In the segmentation module of OSIRIS v4.1, the contours of the iris are detected by the Viterbi algorithm [51]. Subsequently, a modified Daugman’s rubber-sheet model is deployed to perform normalization. With this approach, the original iris image in any resolution is unwrapped into a size-invariant band. In this work, all the iris images are normalized to the size of 103 × 360. To eliminate the inference, we cut off the first 3 rows and the bottom 40 rows and then resize the image to a resolution of 30 × 360. After that, the contrast of the image is enhanced using histogram equalization [52]. In addition, a conditional horizontal cropping step is leveraged in the training stage of full-size network, which crops 20–164 columns and 240–306 columns and hence obtains a 30 × 150 normalized image. It contributes to reducing the time consumption and the probability of overfitting in the training stage.

### 3.2. Online Augmentation Method

**Figure 3:**
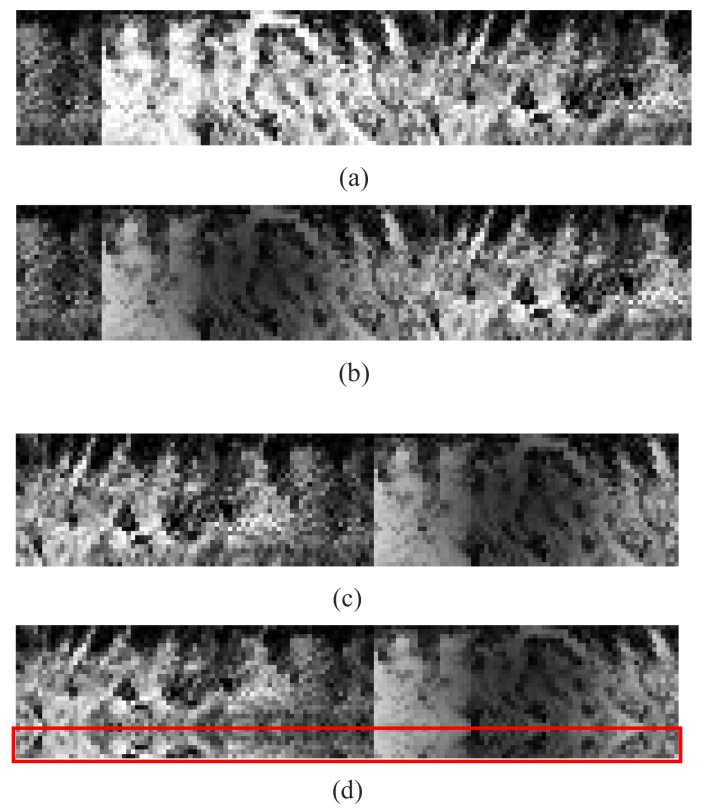
The examples of the output for each different online augmentation layer. (**a**) is a normalized and enhanced iris image randomly picked from CASIA-V3-Interval database. (**b**–**d**) are the example of brightness jitter, horizontal shift, and longitudinal scaling operation, respectively. The red rectangular window shown in (**d**) marks the mirrored part of the iris.

We develop three well-designed augmentation schemes, namely brightness jitter, horizontal shift, and longitudinal scaling, to simulate the variant and contamination of the normalized iris. The explanation for each scheme is expressed as follows:

(1) Brightness jitter: As shown in Figure 3b, a random part of the region is set to be darker than the surrounding area by convoluting the image with a Gaussian kernel $G\in {\mathbb{R}}^{128\times 128}$ with a variance of 28 along both the x-axis and y-axis. The transformation simulates the non-uniform illumination in an iris acquisition environment. Additionally, previous studies proved the feasibility of achieving a performance improvement by covering a random region of the input images [53,54].

(2) Horizontal shift: To overcome the varying rotation degree in various subjects, we perform the translation on normalized iris using a random offset. Figure 3c depicts a sample of horizon shifts in the right direction. According to the definition of the normalized iris image, the horizon shift in normalized iris corresponds to the rotation in the original iris image.

(3) Longitudinal scaling: To better adapt to valid iris size changes caused by individual difference or pupil scaling, a longitudinal scaling augmentation is conducted on normalized iris. In this way, the normalized iris is scaled by a random factor F∈[0.75,1.25] along the longitudinal direction. If F≥1, then the first 30 rows are preserved as a valid region. Otherwise, a number of last certain rows is mirrored to compensate for the original image height. All the image rescaling operations are conducted by nearest-neighbor interpolation. Obviously, the longitudinal scaling refers to the radial scaling in an unsegmented iris. An example of this operation is illustrated in Figure 3d.

Furthermore, two input channels are randomly switched in the training phase. It is worth highlighting that these augmentation operations are performed online, which means the images are stochastically adjusted during the CNN optimization stage, while preserved in the testing phase. To that end, these functions are integrated into an augmentation layer at the front of the CNN architecture, which is performed in each mini-batch of the input data. Benefiting from the online augmentation, only a few classes are needed for model training and finetuning.

### 3.3. Modified 2-ch Deep Convolutional Neural Network

**Figure 4:**
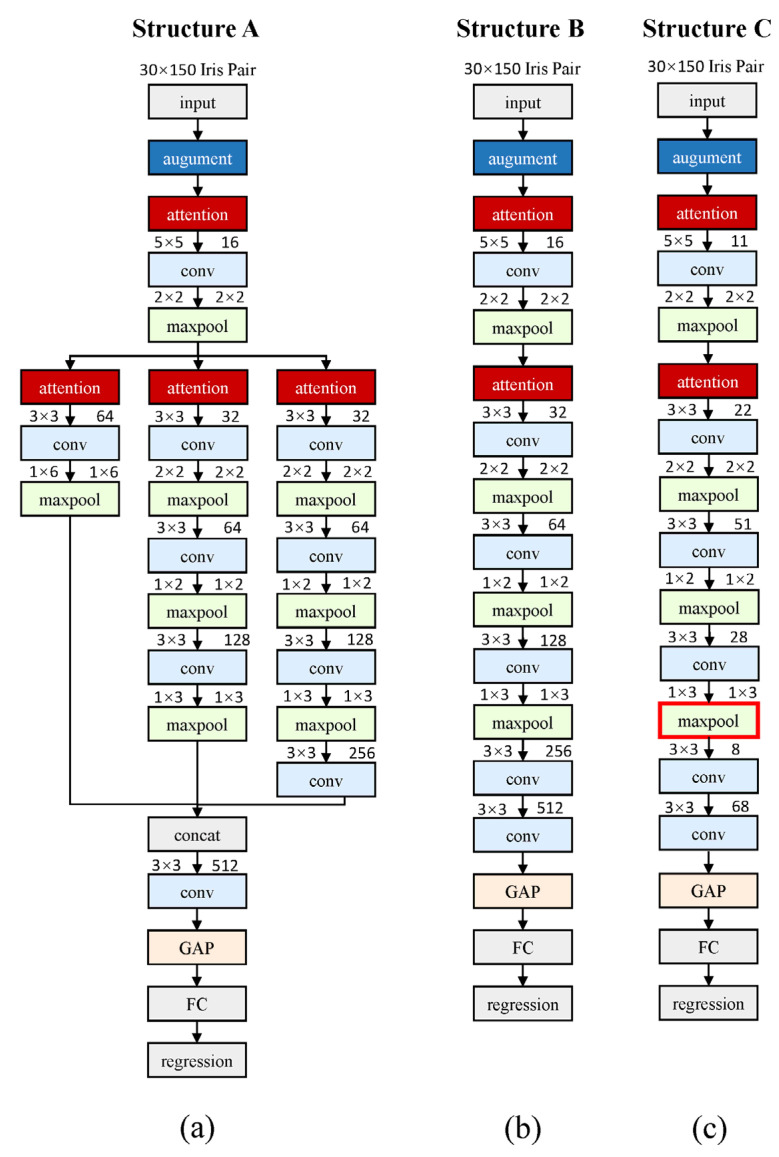
The architecture of the proposed convolutional neural network. (**a**) presents the full-size 2-ch CNN (Structure A). (**b**,**c**) illustrate the branch-pruned and channel-pruned CNN (Structure B and C), respectively. For convenience, a convolutional layer, batch normalization layer, and a ReLu activation layer are integrated into a convolution block (conv) in order. For a specific convolution block, the kernel size and the number of output channels are marked in the upper left and right corners of the box, respectively. All the convolution operations are with the stride of 1 in each direction. Moreover, for the max-pooling layer (maxpool), the pooling region and pooling stride are also marked in the upper left and right corners of the box, respectively.

As mentioned above, the 2-ch CNN method presents great potential in iris recognition. However, the optimal CNN architecture, which can achieve a trade-off between performance and computational complexity, has not been fully explored. In contrast to empirically adjusting the hyperparameters such as the depth of the network and the number of kernels, we employ the pruning method to automatically search for a satisfactory CNN architecture. As shown in Figure 4a, a full-size CNN (Structure A) is first established and trained on the CASIA-V3-Interval training set, which extends a total of three branches with different depths between the first and the last convolutional layer. Hence, three different forward and backward propagation paths are established. The motivation of constructing this full-size architecture is to explore the possibility of capturing and integrating various depth’s feature representation. At the end of the network, a global average pooling (GAP) layer is utilized to replace the fully connected (FC) layer. The GAP operation sums up all elements in each feature map channel regardless of its tensor shape. With this approach, the network can handle any resolution samplings of a normalized iris image [55].

The loss function is the learning objective of a network and, hence, it should be carefully discussed and designed. Since the comparison of paired iris images is more inclined to distance measurement rather than classification, we take MSE with $L_{2}-\mathrm{norm}$ as the loss function. Experiment results indicate that the MSE loss outperforms the cross-entropy (CE) loss and hinge-based loss [40]. The learning objective is the following:

(1) $$\underset{\omega }{\min}\sum_{i=1}^{M}{\left(Y_{i}-T_{i}\right)}^{2}+\frac{\delta }{2}||\omega |{|}_{2}$$

where ω denote weights of the neural network, *M* = 256 stands for the mini-batch size, $Y_{i}\in \mathbb{R}$ is the *i*-th network output value, and $T_{i}\in \{0,2\}$ the corresponding target label (with 0 and 2 denoting a matching and a non-matching pair, respectively). The regularization coefficient δ is set to 0.001 in this study. The $L_{2}$ regularization is motivated to alleviate the overfitting of the model by constraining the summation of squares of all learnable parameters. With an appropriate penalty coefficient and sufficient optimizing iterations, the network can automatically filter out the inoperative weights and retains the informative convolution kernels so as to enhance the generalization performance.

In Figure 4a, it can be noticed that we place a radial attention layer at the head of each branch. Assume the tensor $X\in {\mathbb{R}}^{H\times W\times C}$ is feature map fed into the radial attention layer and the column vector $W_{a}\in {\mathbb{R}}^{H}$ is the radial attention weight to be learned, where *C*, *H*, and *W* correspond to the channel, height, and width of the feature map, respectively. Then, the output of this layer $Z\in {\mathbb{R}}^{H\times W\times C}$ can be expressed as:

(2) $$Z=\mathrm{repmat}\left(W_{a},[1\;W\;C]\right)\circ X$$

where $\mathrm{repmat}\left(W_{a},[1\;W\;C]\right)$ represents the duplication operation in the 2nd and 3rd dimensionalities for *W* and *C* times of $W_{a}$. The operator ∘ corresponds to the Hadamard product. On the other hand, the gradient of $W_{a}$ in back propagation is computed as:

(3) $$\frac{dL}{dW_{a}}=\sum_{W}\sum_{C}\sum_{B}\frac{dL}{dZ}\circ X$$

where *L* indicates the loss function of the network and *B* the mini-batch size in training phase. The radial attention layer weights different regions along the radial direction in the corresponding original iris image. It is proven that this layer can provide better recognition performance and help to prune the model.

### 3.4. Structural Model Pruning

The pruning operation permanently drops the less important weights from the trained model for computational efficiency. The structural model pruning schemes such as branch level pruning and channel level pruning can be easily implemented without extra optimization costs. In this study, the full-size CNN (Structure A) is first trained by 22,000 epochs on only 33 classes (874 genuine pairs + 1748 imposter pairs) in the CASIA-V3-Interval iris database. The model is optimized by the Adam optimizer [56], with an initial learning rate of $5\times 10^{-4}$ decreasing exponentially to $10^{-6}$. It is noteworthy that all weights in full-size CNN are initialized by the Glorot method [57]. After training, we gather the $L_{1}$ norm of all radial layers of a full-size network and compare them with a fixed threshold $T_{prune}=10^{-3}$. Then, the radial layer whose norm cannot reach the $T_{prune}$ and its corresponding branches are discarded. Finally, a branch-selected network is obtained. As depicted in Figure 4b, only the deepest branch is reserved.

**Figure 5:**
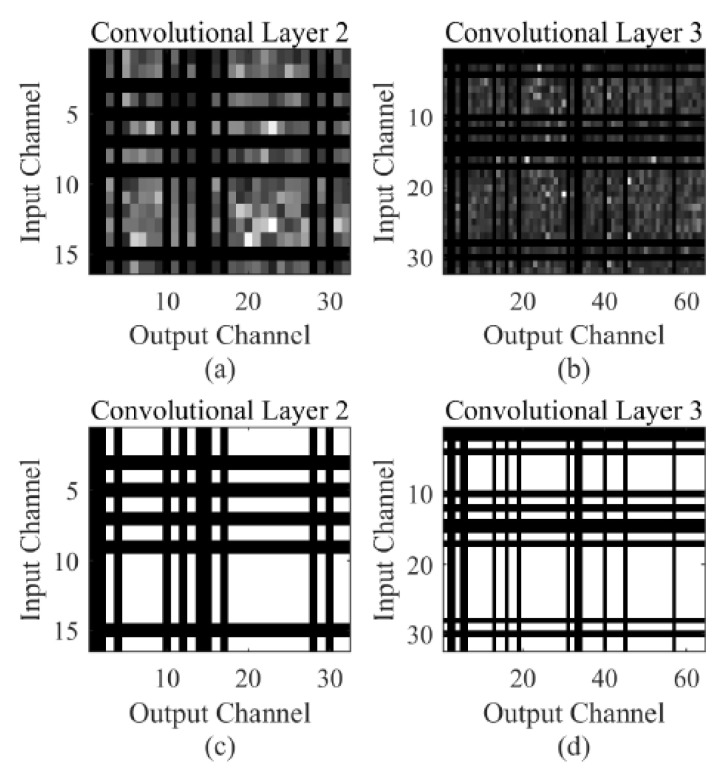
The demonstration of channel level sparsity. Each entry in the matrix represents the $L_{1}$ norm of the kernel. (**a**,**b**) illustrate the 2nd and 3rd convolutional layer channel map. The brighter element represents the more important kernel. (**c**,**d**) are the corresponding pruned channel map. The white regions are reserved while the black regions are discarded.

Additionally, the branch-pruned network can be further condensed by channel pruning. For this purpose, we calculate the accumulated $L_{1}$ norm of the output channel. By applying the aforementioned fixed threshold $T_{prune}$, the unimportant output channels together with their corresponding input channels are cut off permanently. Similarly, the corresponding weights in batch normalization (BN) layers are pruned. The application of $L_{1}$ norm can preserve more useful kernels, which can lead to a less performance loss. Figure 4c depicts the architecture of the final pruned network (Structure C). It is clear that the whole network, especially the last two convolution blocks of the network, has far fewer parameters compared to the network without channel pruning (Structure B). Actually, Structure C only contains 33,268 parameters, which is far smaller than all the CNN architectures employed in previous iris recognition studies to the best of our knowledge. Figure 5 depicts an example of channel pruning. Figure 5a shows the 16 × 32 channel map in the 2nd convolutional layer in branch-pruned 2-ch CNN (Structure B). In this layer, we have a total of 512 convolution kernels. As shown in Figure 5c, the channel map is reduced to a size of 11 × 22. The output channel pruning also leads to the input channel pruning in the next layer, which is presented in Figure 5b,d as horizontal black lines. The 3rd channel map is hence pruned from the size of 32 × 64 to 22 × 51. 

After structural pruning, the model needs to retrain for maintaining accuracy. We retrain the model on the same training set of CASIA-V3-Interval by 500 epochs, with a minor learning rate of $10^{-6}$. Finally, we finetune the model on different target database to adapt the domain of the target database. It is worth emphasizing that this finetuning procedure is done with the non-cropped normalized iris images in resolution of 30 × 360, which enables the model to capture more informative features and gain better performance. Specifically, the model is finetuned for 1200 epochs, with an initial learning rate of $5\times 10^{-5}$ decreasing exponentially to $2\times 10^{-7}$.

### 3.5. Efficient Encoding for Large Scale Iris Recognition

A primary limitation of the 2-ch CNN method lies in its massive computational complexity. When a sample is sent in, this sample is paired with all the samples in the database. Each pair is fed into the 2-ch CNN to conduct forward propagation, which is more time-consuming than only calculating the encoding distance. Accordingly, we propose a hybrid method in the identification scenario. The max-pooling (MP) layer marked with a bold red rectangular frame in Figure 4c acts as an encoding layer. When performing iris registration for large scale iris identification scenario, which is time-sensitive, a pair of identical normalized iris images is fed into the 2-ch CNN and then the flattened feature map of the encoding layer is extracted and stored as a unique encoding for discarding the low confidence sample pairs. Meanwhile, the original normalized iris images are also stored for a more accurate classification with condensed 2-ch CNN. Unlike other deep learning-based feature encoding methods, the 2-ch CNN is not trained for the encoding purpose. However, we find that some of the middle layers in the condensed 2-ch CNN model have a powerful encoding ability, which means we can simultaneously obtain an iris image encoder with lower precision but higher efficiency and a pair-wise iris image classifier with reverse properties by training only one model.

## 4. Experiments and Results

### 4.1. Experimental Iris Databases

Three databases, namely CASIA-V1 [58], CASIA-V3-Interval [59], and CASIA-V4-Thousand [59], are adopted to conduct assessment and analysis in this study. The detailed description of these databases is reported as follows:

(1) CASIA-V1: A total of 756 iris images with a resolution of 320 × 280 were collected from 108 eyes. For each eye, seven images were captured in two sessions, where three samples were collected in the first session and four in the second session. The pupil regions of all iris images in this database were automatically detected and replaced with a circular region of uniform intensity to mask out the specular reflections from the NIR illuminators. Since the iris pupil is edited and the image quality is extremely clear, we conduct the ideal condition experiment using this database.

(2) CASIA-V3-Interval: In this database, a total of 2639 images with a resolution of 320 × 280 were collected from 249 subjects but 395 eyes. The number of gathered images was not fixed for each eye. Therefore, for the convenience of the experiment, only 233 classes (eyes) with an image number of seven or more are selected in this work. If the number of images in a class is greater than seven, we randomly choose seven images. In this study, the first 33 classes are adopted to train the original full-size network.

(3) CASIA-V4-Thousand: As the first publicly released iris database containing a thousand people, a total of 20,000 iris images were included in the CASIA-V4-Thousand database. Thus, ten pictures with a resolution of 640 × 480 were enrolled for each person’s left and right eye. The dominating variations in the database are eyeglasses and strong specular reflections, which pose a more significant challenge to the iris recognition algorithm. We select the left eye of the first 648 subjects to carry out related experiments. In addition, seven pictures are randomly selected from each class.

**Figure 6:**
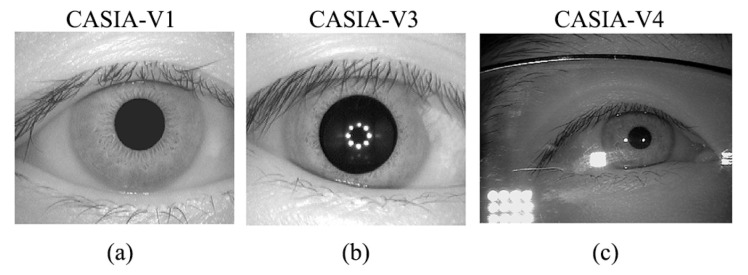
The sample iris images randomly picked from three databases.

For convenience, here we use CASIA-V3 and CASIA-V4 database to represent the CASIA-V3-Interval and CASIA-V4-Thousand database. As shown in Figure 6, a typical sample is randomly picked from each database. It is worth noting that only the training data in the CASIA-V3 dataset are employed to train the original CNN from scratch, and the training data in other databases are used for finetuning.

### 4.2. Experimental Results for Iris Identification and Verification

To fully assess the accurateness and effectiveness of our algorithm, we test the model on three publicly available databases under different configurations. The identification evaluation criterion is designated to the recognition accuracy, which is defined as the ratio of the number of correct recognitions to the total number of recognitions. In an identification epoch, the sample paired with all samples in the database, and if the sample pairs with the lowest score belong to the same class, then it is called a correct recognition. Since each class in all databases uniformly has seven images, in this study, all the reported identification results are the mean of the 7-fold cross-validation. On the other hand, we select the equal error rate (EER) as the evaluation criterion of verification scenario. The evaluation program pairs all possible combinations of the sample in the database and gained the false acceptance rate (FAR) and false rejection rate (FRR) at different thresholds. With a particular threshold, the FAR is equal to FRR and also EER. 

**Figure 7:**
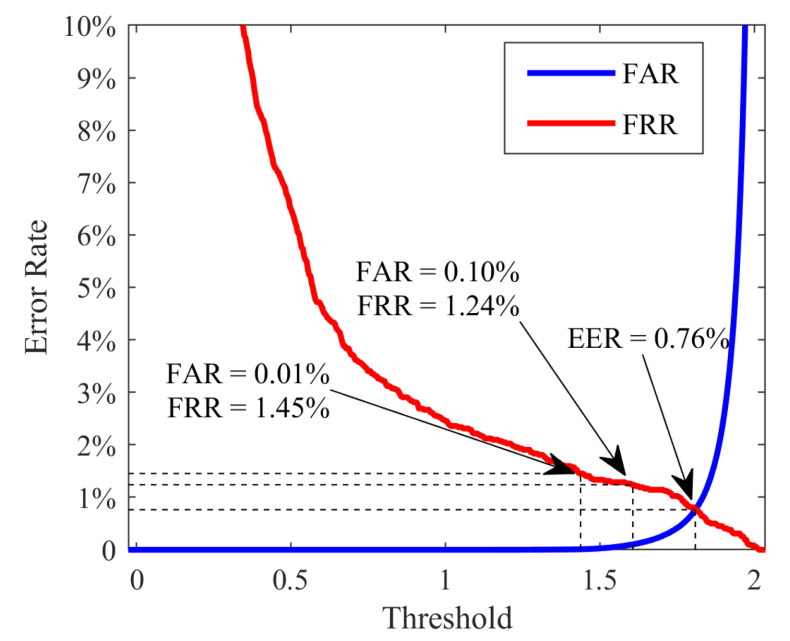
The ROC curve of the proposed algorithm on database CASIA-V3-Interval.

Table 1 illustrates ten comparative experimental results evaluated in the identification and verification scenario. In the identification scenario, two typical conditions, including one picture registered and the six pictures registered, are considered. At the same time, the EER, along with the FRR at FAR = 0.1% and FRR at FAR = 0.01%, is regarded as the important performance indicator for verification assessment. As described above, the proposed network is first trained on 33 classes in the CASIA-V3 database from scratch, and the model is then retrained on the same 33-class training set after pruning operation. The retrained model yields an excellent EER of 0.76%, and outstanding recognition accuracy of 98.95% is fulfilled with only one picture registered in each class. Further, the full receiver operating characteristic (ROC) curve is plotted in Figure 7. For the CASIA-V1 database, the result achieved by the transferred model is reasonably good because of its high image quality. Moreover, if the model is finetuned with 20 classes to adapt its domain, the performance cannot gain much improvement. The CASIA-V4-Thousand database is widely recognized as one of the most challenging iris databases. Therefore, we comprehensively evaluate our proposed algorithm on it. As can be seen from experiments 4–10, various classes are employed for finetuning, and more than 600 classes (approximately 10M pairs) serve as the testing set. Experiment 4 indicates that the transferred model can reach an accuracy of 98.21% in the identification scenario and an EER of 3.54% in the verification scenario. However, the transferred model may not be suitable for the few pictures registered condition or high security required application scenarios. Sequentially, we finetuned the model with 5–30 classes from the target CASIA-V4 database to fit the target domain feature distribution. Interestingly, it can be observed that by exploiting only five classes (138 genuine pairs and 68 imposter pairs) for finetuning, we can gain a significant performance improvement. With the increase of the amount of tuning data, the performance of the model is gradually improved. The results indicate that 30 classes (815 genuine pairs and 1630 imposter pairs) can positively meet the finetuning demands. The adequately finetuned model is able to provide an accuracy of 97.92% to 99.77% in the identification scenario. An extremely low EER of 1.19% can be reached while the FRR is 2.16% and 3.31% under the FAR = 0.1% and FAR = 0.01% criterion. The extraordinary performance of the model ensures it can be applied to iris recognition scenes with high accuracy and security requirements.

### 4.3. Ablation Study

We perform extensive ablation experiments on the CASIA-V3-Interval database to comparatively demonstrate the effectiveness of each technique we employed in this study. From the results illustrated in Table 2, it can be observed that our well-designed model (i.e., the model in experiment 2) defeats the widely used baseline model codenamed as Resnet-18 with an EER improvement of 0.29%. The $L_{2}$ regularization term can not only help to prune the network architecture but also contribute to enhancing the model. According to experiment 4, it can be seen that if the online augmentation layer is removed, the performance of the model will suffer greatly. This can demonstrate the effectiveness and necessity of the online augmentation method for small-scale datasets. The comparison between experiment 5 and experiment 2 shows the superiority of the MSE loss over the CE loss. Experiment 6 indicates that the radial attention layer can reduce EER at the cost of adding very few learnable parameters. From experiments 6, 7, and 8, we can conclude that if we only prune the model without the retraining procedure, the accuracy of the model will decrease. When performing the finetuning, the performance of the pruned model will exceed that of the unpruned model. In experiment 9, we specially build a single branch network with the same structure as the pruned network and train from scratch. Compared with the result in experiment 8, the necessity of pruning is obvious. Finally, we add the reshape operation, which means the model is trained on a cropped normalized iris image dataset with a resolution of 30 × 150 but finetuned by an uncropped dataset with a resolution of 30 × 360. In this way, despite some interferences mixed in, more iris textures can be captured, and hence the best performance is gained.

To better examine the effectiveness of the proposed online augmentation scheme, an ablation study is done on 150 testing classes and five finetuning classes in CASIA-V4. As shown in Table 3, all three types of augmentation schemes can effectively contribute to improving the performance of the model. Compared to the non-augmented situation, scheme BJ., HS., and LS. alone can offer a 0.16%, 0.30%, and 0.09% reduction in EER, respectively. Moreover, the combination of these three online augmentation methods can achieve a better result. When all three augmentation schemes are utilized, the best performance is yielded.

### 4.4. Encoding Ability Research

Now we explore the encoding ability of the 2-ch CNN model. For convenience, we define the discarding accuracy *D-Accuracy* as the following equation:

(4) $$D-Accuracy=\frac{\sum_{i}^{N_{d}}\sum_{j}^{N_{R}}1\{S_{ij}<Thr\}}{N_{d}\times N_{R}}$$

where *Thr* is a threshold, which can be specified manually or by setting the discarding ratio. $N_{d}$ and $N_{R}$ are the number of identification epochs and the number of registered iris images in each class, respectively. $S_{ij}$ is introduced as the matching score of pairing the original sample with its intra-class *j*-th sample in the *i*-th identification epoch. The descriptor 1{⋅} stands for the indicator function. In this experiment, the discarding accuracy is defined to evaluate the encoding performance of the model. We traverse the encoding capabilities of all layers on CASIA-V3 database. As depicted in Table 4, the discarding accuracy of each layer and its corresponding feature length and matching time in one identification epoch is analyzed in detail. It can be observed that layer 17 reaches the best discarding accuracy and the smallest variance simultaneously. Nevertheless, it is not the best choice for encoding matching due to its corresponding lengthy feature vectors and high time consumption. There are two feasible choices for generating encodings. First is layer 19: compared to the 17th layer, the 19th layer has more than doubled the time-consuming reduction with a loss of discarding accuracy of approximately 1%. The second choice is layer 22, which can achieve 90.23% discarding accuracy even if the feature length is only 960. In this work, we choose the 19th layer as the encoding layer.

**Figure 8:**
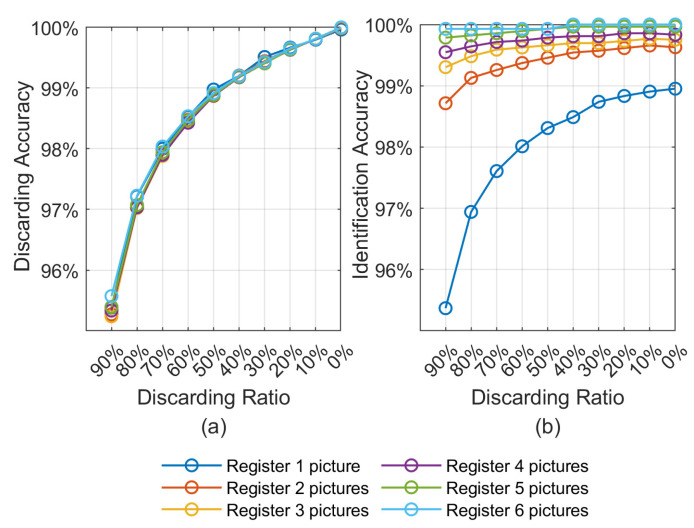
(**a**) The discarding accuracies under different discarding ratio and different number of registered pictures. (**b**) The identification accuracies under different discarding ratio and different number of registered pictures.

Figure 8a demonstrates the discarding accuracy of the proposed modified 2-ch CNN model in different discarding ratios ranging from 0% to 90% and a different number of registered pictures ranging from 1 to 6. We can conclude that a discarding accuracy over 95% can be achieved with a 90% discarding ratio regardless of the number of registered images per class. Such a satisfying result demonstrates the feasibility of taking the 2-ch CNN as a feature extractor. Besides, Figure 8b plots the curve of identification accuracy varying with the discarding ratio under a different number of registered pictures. It reveals the effectiveness of the discarding process for the identification scenario. Meanwhile, the impact of different number of registered pictures on identification accuracy is also well demonstrated. We can see that when only one picture is registered per class, the identification accuracy is mainly restricted by the discarding accuracy. However, when more than two pictures are registered in each class, the identification accuracy is almost independent of the discarding accuracy. Even in some cases, the screening process can contribute a slight improvement to identification accuracy. This experimental result indicates that only three pictures of each eye are needed to ensure an identification accuracy of more than 99%.

## 5. Discussion

### 5.1. Weight Visualization

**Figure 9:**
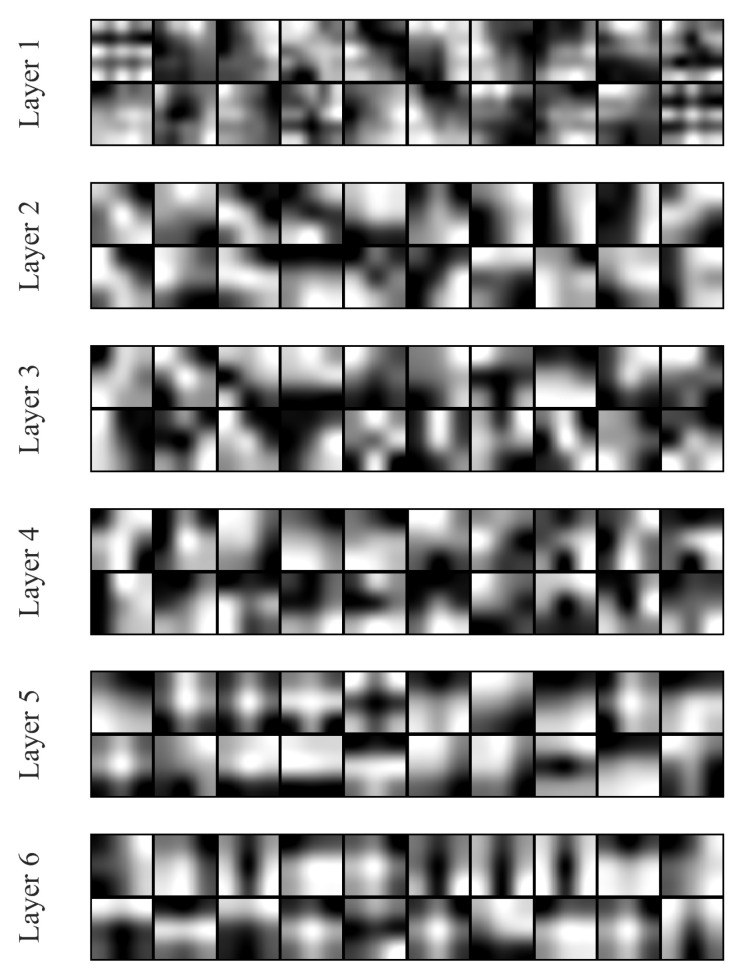
The visualization of the convolution kernels randomly picked from each convolution layer. Each kernel in size of 3 × 3 or 5 × 5 is resized to a higher resolution by cubic interpolation.

We further visualized some examples of the 2D convolution kernel at the microscopic level learned from each convolution layer. It can be seen in Figure 9 that the convolution kernels in the first four layers learn somewhat chaotic kernel maps. Using these kernels, the model can adapt to various inputs with stochastic perturbations and thereby gain the feature in abstract and robustness. On the other hand, the kernel map of the last two layers seems more specific and regular. Such a phenomenon may be because the feature maps processed from the previous layer have been regularly reshaped, which is insensitive to input disturbances.

**Figure 10:**
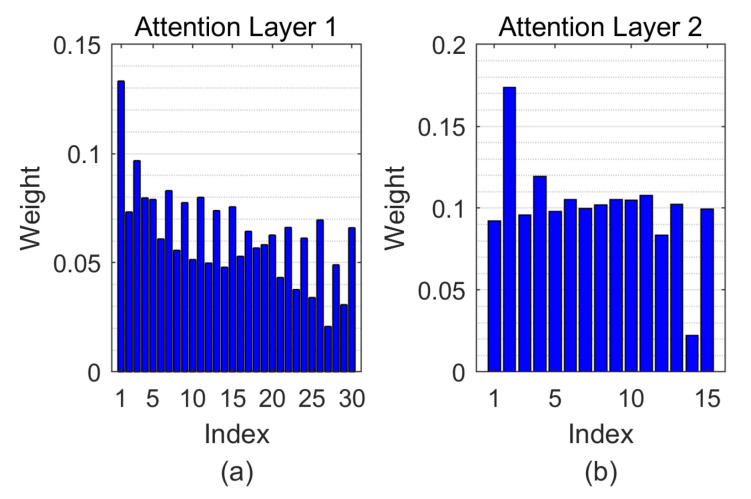
The visualization of the radial attention layer in proposed CNN architecture. (**a**,**b**) correspond to the attention weight for radial attention layer 1 and 2, respectively.

As aforementioned, the radial attention layer acts as a branch selection gate as well as an iris region weight function. Figure 10 illustrates the weights that the radial attention layers learned. The smaller value of the X-axis stands for the radial part closer to the pupil. It can be seen from the weight distribution of both radial attention layers that the closer to the pupil, the larger the weight.

### 5.2. Time Consumption Experiments

**Figure 11:**
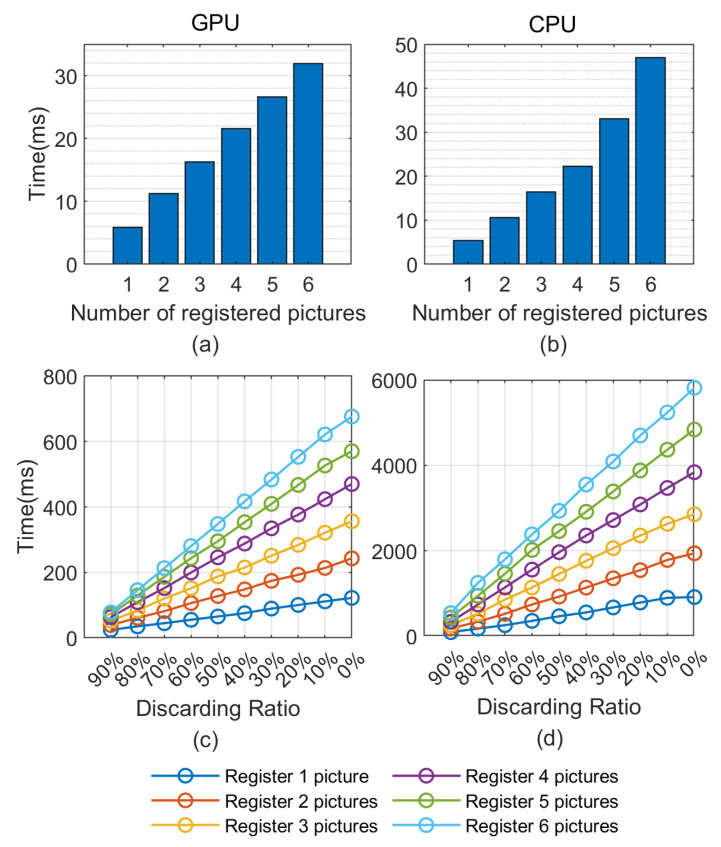
The summary of the time consumption in different conditions. (**a**,**b**) correspond to the time consumption of screening procedure achieved by GPU and CPU, respectively. (**c**,**d**) the time consumption of identification procedure achieved by GPU and CPU, respectively.

In order to further ascertain the effectiveness of the encoding matching process, we calculate the time consumption of the algorithm under different parameter configurations and different devices, which are shown in Figure 11. Traditional iris recognition algorithms are usually deployed on the central processing unit (CPU) [60], and they are hard to parallelize. By contrast, CNN can easily be parallelized and deployed on the graphics processing unit (GPU) by using the mainstream deep learning framework [61]. To comprehensively evaluate the proposed algorithm’s execution efficiency in different application situations, GPU (NVIDIA GTX-1080) and CPU (i7-8700K, 3.7GHz) are considered in this experiment. This experiment is also conducted on CASIA-V3-Interval database. An interesting phenomenon can be observed from Figure 11a,b that with the increase of the number of registered pictures in each class, the computational time consumption of the encoding matching process grows linearly on GPU and nonlinearly on CPU. This may be caused by different caching mechanisms in the CPU. The elapsed time in identification procedure represented in Figure 11c,d can correspond to the identification accuracy in Figure 8b under the same conditions. For GPU devices, it takes only 24 ms (with 90% discarding ratio and one picture registered per class) to 677 ms (with no discarding and six pictures registered per class) to recognize 1400 images. In contrast, for CPU devices, it takes 95 to 5816ms under the same conditions. It should be specified that since the 2-ch CNN network is affected by the stack order of the input channels, we conducted double forward propagation with different channel orders. About 0.25 ms is needed for our model to process one pair of images, which is nearly three times less time-consuming than the DeepIris model mentioned in the literature [47]. Based on this premise, we can choose the corresponding device and discarding ratio according to the actual demand to balance the efficiency and expenses.

### 5.3. Interpretability Analysis

**Figure 12:**
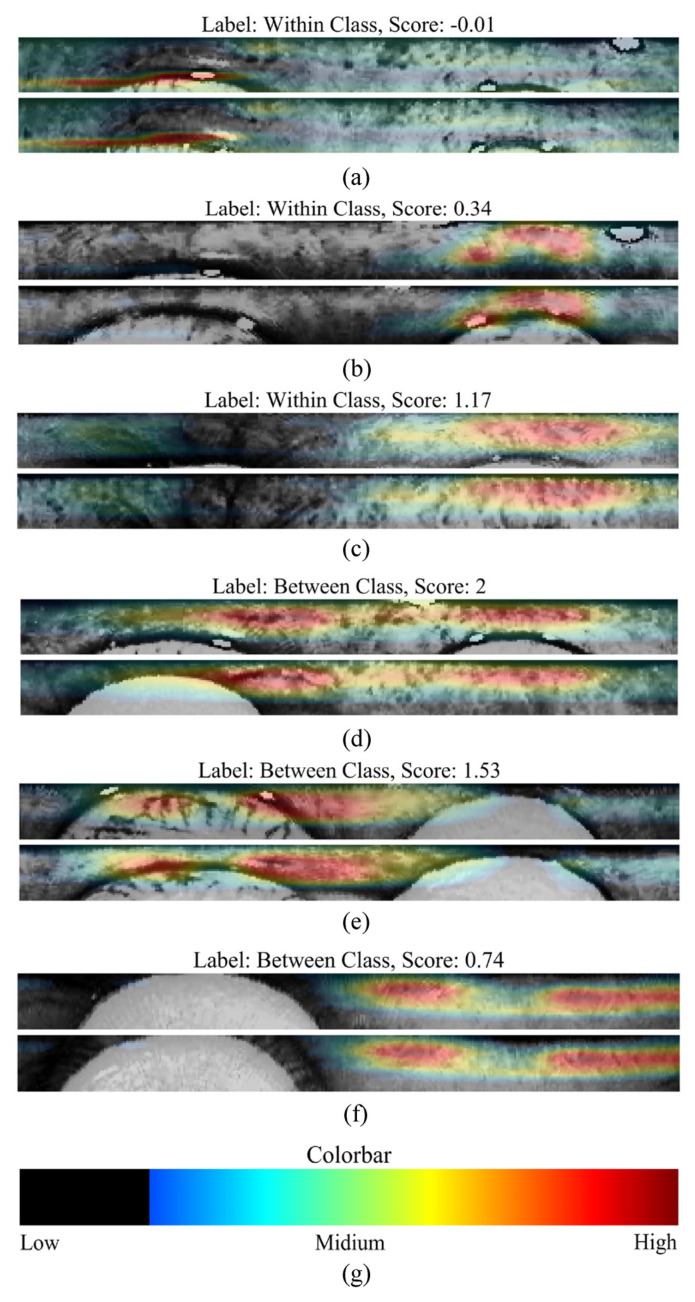
(**a**–**f**) are the heat maps of ROI visualized by Grad-CAM algorithm. The most discriminative iris texture area is marked by red and yellow. (**g**) is the colorbar of the heat map, where the yellow and red area represents a higher score while the green and blue area correspond to the medium score, and the bottom 20% score is set to zero.

Despite the 2-ch CNN method has proven its effectiveness in image patch comparison, no efforts have been made to understand how the model obtained its score. In this study, we employed the gradient of the model regression output with respect to the last convolutional layer in a network to find which parts of the iris dominate the output score. The Grad-Cam approach [62] is deployed for this purpose, and the analysis results are shown in Figure 12. The red part of the image in both two channels can be regarded as the region with the most distinctive iris texture. Figure 12a,d demonstrate two typical cases of ideal recognition. For the iris pair identified as intra-class, only a few areas are marked in red, while for the iris pair between classes, most regions are considered inconsistent. In addition, two situations in which the confidence of the output score is relatively low are depicted in Figure 12b,e. We can see that contaminations, including eyelids and eyelashes, are enrolled in these normalized iris images, which affects the judgment of the model. At last, we provide two examples of false detection in Figure 12c,f. The false-negative in Figure 12c is caused by the uneven width of the segmented iris image, which may be the result of pupil dilation generated with dramatic light changes or other interferences. Correspondingly, a false-positive is described in Figure 12f. We observe that this iris pair is too similar and that it can be easily mistakenly identified even by human visual inspection. On considering all these images, it can be found that the different parts marked by our model are seldomly located in the polluted area, which is adequate proof that the model has strong robustness to the stochastic contaminations in the iris image.

### 5.4. Comparison of Iris Recognition Results

Table 5 compares our proposed method with other methods assessed on CASIA databases in recent years. For the classic iris recognition algorithm, the comparison is made with IrisCode. Othman et al. [50] constructed the OSIRIS framework and presented the classic iris recognition chain, which reproduced the IrisCode algorithm proposed by Daugman [29]. The IrisCode is a handcrafted feature, but it can be applied to the new database without training data. Our proposed method fulfills an EER of 3.54% in the cross-database case on the CASIA-V4 dataset, which is approximately the same as the IrisCode method. However, when a few samples are utilized for finetuning the model, a significant performance advantage is shown by our method. For the deep learning-based method, the encoding ability of the off-the-shelf CNN features are explored in No. 2, 3, 7 entries of Table 5. Moreover, in examples from the literature [45,63,64,65], new CNN models were established, which were trained from scratch, and then their classification ability investigated. It can be seen that all the mentioned methods need a great number of samples for training, which is impossible for practical application scenarios. Moreover, the CNN models utilized in previous studies also have far more parameters, while the performance is evidently lower than ours. The 2-ch CNN-based method was studied in more recent research [49] and [66]. Proença et al. [49] proposed a segment-less CNN model based on VGG-19, and the model was trained by 45,000 genuine pairs and 1 million imposter pairs on CASIA-V4. As a result, an EER of 3% was acquired on testing phase, which was much lower than ours. Chen et al. [66] proposed a novel loss named Tight Center and assessed it with three types of classic architectures on the CASIA-V4 database by adopting a cross-database scheme. As a result, the best result with an EER of 2.36% and an accuracy of 99.58% was reported, which was slightly better than our cross-database results. However, their method was trained on 50,632 images on the ND-IRIS-0405 iris database, and then the model was evaluated on 38,573 genuine pairs and 107,589 imposter pairs on the CASIA-V4 database. In contrast, our method is trained on only 231 images in the CASIA-V3 iris database and test on more than 10M pairs in the CASIA-V4 database. Moreover, we compute the number of parameters and computational cost of our model and other compared models. As illustrated in Table 5, our model has a total number of 33K parameters and floating-point operations (FLOPs) of 49.1M, which are lower than most of the previous models. The model employed in [64] has the least computational cost, but its identification accuracy is far lower than ours. Overall, our proposed condensed 2-ch CNN method achieves state-of-the-art performance on three publicly available databases with few sample tuning and much fewer model parameters.

## 6. Conclusions

This work presents a new framework for large-scale iris verification and identification using 2-ch CNN. Four key innovations, including the hybrid framework for large-scale iris identification and verification, radial attention layer for weighting different iris regions, online augmentation schemes for enhancing the robustness, and structural pruning for alleviating computational burden, are introduced in 2-ch CNN to improve the performance. The proposed method is evaluated on three publicly available databases. The experimental results indicate that our method has outstanding efficiency and performance over previous studies utilizing deep learning-based methods and handcrafted feature-based methods. Moreover, the satisfying results achieved on the CASIA-V4-Thousand database indicate that the proposed method can be applied in challenging iris recognition situations. This work also investigates the encoding ability of the 2-ch CNN and finds that some middle layers have excellent encoding ability. This enables the 2-ch CNN to be applied to large-scale iris identification. Since all three types of online augmentation schemes carefully designed in this study are proven to be beneficial for model performance, we will continue to develop these schemes and consider more contaminations in iris images, such as eyelids and eyelashes. Additionally, the multi-modal identification approach, which combines iris recognition with other biometrics approaches such as face recognition and palmprint recognition, is suggested for future work.

## Figures and Tables

**Table 1:** The identification and verification results for different database using different model configuration.

No.	Database (Method)	Training Classes	Testing Classes	Identification		Verification		
				Register 1 Picture	Register 6 Pictures	EER	FRR@ FAR = 0.1%	FRR@ FAR = 0.01%
1	CASIA-V3	33	200	98.95%	100%	0.76%	1.24%	1.45%
2	CASIA-V1	0	108	99.51%	100%	0.35%	0.57%	1.32%
3	CASIA-V1	20	88	99.76%	100%	0.33%	0.43%	1.46%
4	CASIA-V4	0	648	89.53%	98.21%	3.54%	16.92%	31.13%
5	CASIA-V4	5	615	94.89%	99.47%	2.20%	5.84%	12.40%
6	CASIA-V4	10	615	96.10%	99.58%	1.80%	4.28%	8.87%
7	CASIA-V4	15	615	97.12%	99.72%	1.30%	2.89%	5.40%
8	CASIA-V4	20	615	97.65%	99.79%	1.23%	2.39%	3.96%
9	CASIA-V4	25	615	97.86%	99.81%	1.18%	2.25%	3.50%
10	CASIA-V4	30	615	97.92%	99.77%	1.19%	2.16%	3.31%

**Table 2:** The ablation study under different model configuration.

No.	Structure	Loss	Reg.	Aug.	Att.	Pru.	Fin.	EER
1	Resnet	MSE	√	√	×	×	×	1.55%
2	A	MSE	√	√	×	×	×	1.26%
3	A	MSE	×	√	×	×	×	1.86%
4	A	MSE	√	×	×	×	×	3.40%
5	A	CE	√	√	×	×	×	1.55%
6	A	MSE	√	√	√	×	×	1.09%
7	C	MSE	√	√	√	√	×	1.22%
8	C	MSE	√	√	√	√	√	1.03%
9	C ^†^	MSE	√	√	√	√	√	1.26%
10	C ^o^	MSE	√	√	√	√	√	0.76%

Reg., Aug., Att., Pru., and Fin. are the abbreviation of regularization term, augmentation layer, attention layer, pruning, and finetuning. † The model is trained from scratch. ^o^ The uncropped iris images with resolution of 30 × 360 serve as input.

**Table 3:** The ablation study of the online augmentation method.

No.	BJ.	HS.	LS.	EER	FRR@ FAR = 0.1%	FRR@ FAR = 0.01%
1	×	×	×	2.80%	9.46%	17.05%
2	√	×	×	2.64%	8.00%	14.86%
3	×	√	×	2.50%	8.89%	16.83%
4	×	×	√	2.71%	9.30%	17.43%
5	×	√	√	2.36%	7.84%	16.16%
6	√	×	√	2.50%	6.48%	14.41%
7	√	√	×	2.36%	7.84%	14.48%
8	√	√	√	2.27%	6.06%	13.97%

BJ., HS., and LS. stand for the brightness jitter, horizontal shift, and longitudinal scaling.

**Table 4:** The comparison of the encoding ability in different depth of the proposed CNN.

Layer	Type	Discarding Accuracy (%)	Feature Length	Time (ms)
1	Input	85.61 ± 2.43	21,600	21.00
2	Attention	85.35 ± 2.61	21,600	20.76
3	Convolution	77.65 ± 2.86	118,800	104.56
4	BN	72.37 ± 3.24	118,800	104.09
5	ReLu	72.19 ± 3.18	118,800	104.09
6	MP	75.32 ± 2.92	29,700	27.57
7	Attention	73.36 ± 3.21	29,700	28.48
8	Convolution	71.37 ± 3.17	59,400	52.91
9	BN	70.82 ± 3.23	59,400	53.02
10	ReLu	76.00 ± 3.26	59,400	53.74
11	MP	83.58 ± 2.68	15,840	15.67
12	Convolution	90.79 ± 1.30	36,720	33.68
13	BN	91.70 ± 0.95	36,720	33.37
14	ReLu	89.48 ± 1.83	36,720	33.28
15	MP	95.88 ± 0.86	18,360	18.31
16	Convolution	96.52 ± 0.70	10,080	10.99
17	BN	96.55 ± 0.68	10,080	10.82
18	ReLu	96.27 ± 1.09	10,080	10.90
19	MP	95.42 ± 1.33	3360	5.00
20	Convolution	80.92 ± 2.66	960	2.25
21	BN	84.39 ± 3.07	960	2.25
22	ReLu	90.23 ± 1.92	960	2.41
23	Convolution	77.75 ± 2.57	8160	9.08
24	BN	77.48 ± 2.22	8160	9.10
25	ReLu	72.51 ± 2.50	8160	9.04
26	GAP	26.79 ± 1.75	68	1.33
27	FC	16.83 ± 1.23	1	1.05

Attention refers to the radial attention layer.

**Table 5:** Comparison on the performance of different methods proposed in recent years.

No	Studies	Year	Method	Parameters/FLOPs	Evaluation Protocol	Augmentation	Result
1	Othman et al. [50]	2016	IrisCode (2D-Gabor filter + Hamming Distance)	-/-	CASIA-V4: 602 classes (for testing)	None	CASIA-V4: 3.5% (Verification EER)
2	Nguyen et al. [43]	2017	Pre-trained CNN (Dense-Net) + SVM	-/-	CASIA-V4: 1000 classes (Train: 70%, Test: 30%) *	None	CASIA-V4: 98.8% (Identification Accuracy)
3	Alaslani et al. [67]	2018	Pre-trained CNN (Alex-Net) + SVM	41 M/2.2 B	CASIA-V1: 60 classes CASIA-V3: 60 classes CASIA-V4: 60 classes (Train: 70%, Test: 30%) *	None	CASIA-V1: 98.3% CASIA-V3: 89% CASIA-V4: 98% (Identification Accuracy)
4	Wang et al. [63]	2018	MiCoRe-Net	>1.4 M/>50 M	CASIA-V3: 218 classes (Train: 1346 images, Test: 218 images) * CASIA-V4: 1000 classes (Train: 9000 images, Test: 1000 images) *	Rotation and Cropping	CASIA-V3: 99.08% CASIA-V4: 88.7% (Identification Accuracy)
5	Tobji et al. [64]	2019	FMnet	15 K/10 M	CASIA-V4: 1000 classes (Train: 70%, Test: 30%) *	None	CASIA-V4: 95.63% (Identification Accuracy)
7	Boyd et al. [68]	2019	Pre-trained/Finetuned CNN (ResNet-50) + SVM	25 M/5.1 B	CASIA-V4: 1000 classes (Train: 70%, Test: 30%) *	None	CASIA-V4: 99.03% (Identification Accuracy)
6	Liu et al. [45]	2019	Fuzzified image + Capsule network	>4 M/-	CASIA-V4: 1000 classes (Train: 80%, Test: 20%)	None	CASIA-V4: 83.1% (Identification Accuracy)
8	Lee et al. [65]	2019	Deep ResNet-152 +Matching distance	>53 M/>10 B	CASIA-V4: 1000 classes (Train: 50%, Test: 50%)	Translation and Cropping	CASIA-V4: 1.33% (Verification EER)
9	Proença et al. [49]	2019	VGG-19 based CNN	138 M/-	CASIA-V4: 2000 classes (Train: 1000 classes, Test: 1000 classes)	Scale transform and Intensity transform	CASIA-V4: 3.0% (Verification EER)
10	Chen et al. [66]	2020	Tiny-VGG based CNN	>10 M/>1.3 B	CASIA-V4: 140 K pairs (Train: 50,632 images on another database)	Contrast, Brightness, and Distortion	CASIA-V4: 99.58% (Identification Accuracy) CASIA-V4: 2.36% (Verification EER)
11	Proposed Method	2021	Condensed 2-ch CNN	33 K/49.1 M	CASIA-V1: 108 classes (Finetune: 20 classes, Test: 88 classes) CASIA-V3: 233 classes (Train: 33 classes, Test: 200 classes) CASIA-V4: 648 classes (Finetune: 30 classes, Test: 615 classes)	Brightness jitter, Horizontal shift, and Longitudinal scaling (Online)	CASIA-V1: 100% CASIA-V3: 100% CASIA-V4: 99.77% (Identification Accuracy) CASIA-V1: 0.33% CASIA-V3: 0.76% CASIA-V4: 1.19% (Verification EER)

* Training set and testing set share same classes. K-Kilo, M-Million, B-Billion.

## Data Availability

The data presented in this study are available in http://biometrics.idealtest.org/ (accessed on 21 October 2019).
